# Attenuated Recombinant Influenza A Virus Expressing HPV16 E6 and E7 as a Novel Therapeutic Vaccine Approach

**DOI:** 10.1371/journal.pone.0138722

**Published:** 2015-09-18

**Authors:** Christoph Jindra, Bettina Huber, Saeed Shafti-Keramat, Markus Wolschek, Boris Ferko, Thomas Muster, Sabine Brandt, Reinhard Kirnbauer

**Affiliations:** 1 Laboratory of Viral Oncology (LVO), Division of Immunology, Allergy and Infectious Diseases (DIAID), Department of Dermatology, Medical University of Vienna, Vienna, Austria; 2 Research Group Oncology (RGO), Equine Clinic, Veterinary University of Vienna, Vienna, Austria; 3 Bluesky Vaccines, Vienna, Austria; Georgia State University, UNITED STATES

## Abstract

Persistent infection with high-risk human papillomavirus (HPV) types, most often HPV16 and HPV18, causes all cervical and most anal cancers, and a subset of vulvar, vaginal, penile and oropharyngeal carcinomas. Two prophylactic virus-like particle (VLPs)-based vaccines, are available that protect against vaccine type-associated persistent infection and associated disease, yet have no therapeutic effect on existing lesions or infections. We have generated recombinant live-attenuated influenza A viruses expressing the HPV16 oncogenes E6 and E7 as experimental immunotherapeutic vaccine candidates. The influenza A virus life cycle lacks DNA intermediates as important safety feature. Different serotypes were generated to ensure efficient prime and boost immunizations. The immune response to vaccination in C57BL/6 mice was characterized by peptide ELISA and IFN-γ ELISpot, demonstrating induction of cell-mediated immunity to HPV16 E6 and E7 oncoproteins. Prophylactic and therapeutic vaccine efficacy was analyzed in the murine HPV16-positive TC-1 tumor challenge model. Subcutaneous (s.c.) prime and boost vaccinations of mice with recombinant influenza A serotypes H1N1 and H3N2, followed by challenge with TC-1 cells resulted in complete protection or significantly reduced tumor growth as compared to control animals. In a therapeutic setting, s.c. vaccination of mice with established TC-1 tumors decelerated tumor growth and significantly prolonged survival. Importantly, intralesional vaccine administration induced complete tumor regression in 25% of animals, and significantly reduced tumor growth in 50% of mice. These results suggest recombinant E6E7 influenza viruses as a promising new approach for the development of a therapeutic vaccine against HPV-induced disease.

## Introduction

Cervical cancer (CxC) is the second most common cause of cancer deaths in women worldwide, with about 500,000 new cases per year of which about 50% are lethal. Persistent infection with mucosal human papillomavirus (HPV) high-risk types, most often HPV16 and 18 (as well as types HPV31, 33, 45 and others) is the major cause for the development of cervical intraepithelial neoplasia (CIN) [1], invasive CxC, and a subset of vulvar, vaginal, penile, and also oropharyngeal cancers. The World Health Organization (WHO) estimates that more than 500 million people are infected by genital HPV types, corresponding to a worldwide prevalence of 9–13% [2]. Genital warts (condylomata) have an estimated prevalence of 1% and arise from low-risk genital HPV infection (90% by HPV6, 11). The two licensed prophylactic bi- or quadrivalent HPV vaccines are highly effective in preventing new infections and associated disease. However, vaccine efficacy is restricted to the two (HPV16, 18) or four (HPV6, 11, 16, 18) included VLP types [3]. In many countries, vaccination coverage remains low, primarily because of financial constraints. Therefore, and given the lifetime cumulative incidence of genital HPV infection of ≥50%, HPV-associated diseases are expected to remain a significant health problem particularly in women, but also in immunosuppressed patients and men who have sex with men (MSM) [2].

Currently, there exists no effective antiviral drug for eradication of persistent HPV infections and induced disease. Current treatment options are primarily ablative or destructive, have significant side effects and prove ineffective in late-stage disease. Immunotherapeutic vaccination is regarded as a promising new tool for the treatment of HPV-induced neoplasia [4]. The viral oncogenes E6 and E7 are constitutively expressed by infected tumor cells and responsible for the maintenance of the malignant phenotype [5,6,7]. Consequently, E6 and E7 are regarded as preferred tumor-specific target antigens. Data from several animal and clinical trials substantiate the therapeutic potential of such an approach [8]. The use of live-attenuated influenza A viruses constitutes a novel strategy for the prevention of influenza A infection [9,10,11]. Genetically manipulated live-attenuated influenza viruses can serve as vaccine vectors to fight a variety of pathogens such as *Mycobacterium tuberculosis* or human immunodeficiency virus (HIV) [12,13,14,15]. Influenza A contains a segmented negative-strand RNA genome assuring viral transcription and replication. The smallest segment codes for two non-structural viral proteins, the interferon (IFN) antagonizing non-structural protein 1 (NS1) and the nuclear export protein (NEP). By blocking induction of type I IFN, NS1 prevents cells from acquiring an antiviral state [16]. Consequently, deletions within the NS1 open reading frame (ORF) lead to suppression of the NS1 IFN inhibitory function and hence to live attenuation of influenza A viruses [17]. NS1 is also suitable for genetic manipulation [18,19,20,21,22]. The truncated NS1 ORF has been shown to tolerate insertion of long foreign sequences. Furthermore, NS1 is highly expressed, thus encouraging immune responses against co-expressed foreign antigens [16]. Immunization of animals with NS1-deleted recombinant influenza virus vectors has been shown to elicit a strong CD8^+^ T cell response against transgenes when using different serotypes in a prime and boost regimen [13,22]. As important safety feature, no DNA intermediates are produced during the influenza A viral life cycle, thus precluding integration into the host genome [23].

Herein, the prophylactic and therapeutic potential of recombinant delNS1 influenza virus vectors expressing HPV16 E6-E7 fusion transgenes was analyzed in an established murine HPV16 TC-1 tumor model [24].

## Results

### Recombinant 16E6E7 and 16E6E7m influenza A Viruses Are Stable and Express Foreign Genes

**Fig 1:**
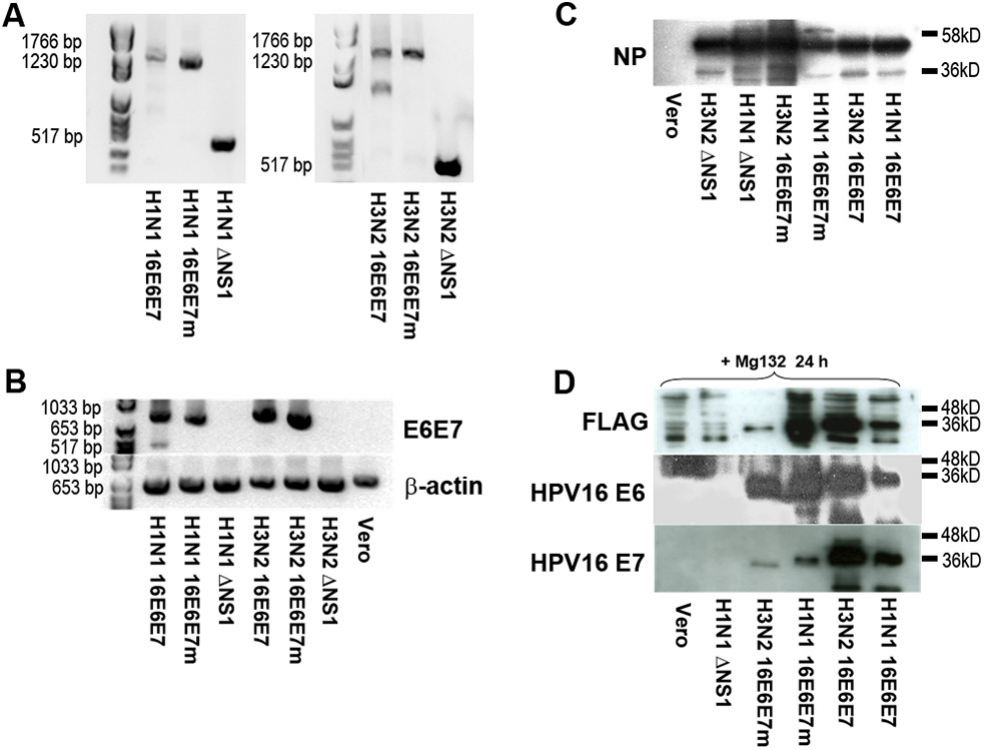
Characterization of recombinant 16E6E7 and 16E6E7m influenza viruses for transgene stability, infectivity and fusion gene expression. Following generation of recombinant H1N1 or H3N2 16E6E7/16E6E7m influenza A viruses and passaging for at least five cycles, **A** whole viral RNA was isolated or **B** Vero cells were infected with 10^5^ pfu of recombinant viruses for 12 hours and whole RNA isolated. Viral or cellular RNA was subjected to RT-PCR analysis amplifying either the whole delNS1-E6E7 ORF (**A**) to analyze stability, or the E6E7 transgene (**B**) to assess fusion gene expression. **C,D** Vero cells were infected for 12 hours in the presence (**D**) or absence (**C**) of proteasome inhibitor MG-132 with H1N1 or H3N2 recombinant or parental influenza viruses as indicated, and whole cell lysates were separated by 10% SDS-PAGE. Viral NP protein (**C**), FLAG (upper panel), HPV16 E6 (middle panel) and E7 expression (lower panel) were assessed using specific monoclonal antibodies (**D**).

NS1-deleted (delNS1) recombinant influenza A H1N1 and H3N2 viruses harboring wild-type (wt) or mutation-inactivated (m) HPV16 E6E7 fusion genes in the delNS1 ORF were obtained by genetic engineering as described below (S1 Fig). To ensure that fusion of initial 11 codons of delNS1 with the E6E7 or E6E7m fusion genes was genetically stable, recombinant viruses were passaged for ≥5 rounds. Total viral RNA was isolated and analyzed for delNS1-E6E7 transcripts by RT-PCR (Fig 1A) and subsequent DNA sequencing. Results confirmed that the delNS1-E6E7 fusion ORFs were retained. Viral titers obtained were comparable to those of parental delNS1 virus controls.

To verify fusion gene expression and infectivity of recombinant viruses, Vero cells were infected with recombinant H1N1 and H3N2 16E6E7 or 16E6E7m influenza viruses or corresponding parental delNS1 strains as controls. After 12h, cells were screened for expression of the structural influenza viral nucleoprotein NP by Western blot. As shown in Fig 1C, cells were as efficiently infected by all 4 recombinant virus types as by parental delNS1 viruses. Expression of E6E7 mRNA was detected in cells infected with H1N1 and H3N2 16E6E7 and 16E6E7m viruses, but not in control cells, i.e. cells infected with parental viruses or uninfected cells (Fig 1B). HPV16 E6E7 fusion protein expression was seen in cells infected with either H1N1 or H3N2 16E6E7 or 16E6E7m viruses (Fig 1D), but not in control cells.

Both fusion proteins were identified as bands migrating at ~38 kD (with 16E6E7m fusion protein migrating slightly faster) when using specific antibodies to HPV16 E6, E7, or FLAG. However, E6E7 and E6E7m proteins were detected only in Vero cells treated with proteasome inhibitor MG132 before and during infection, suggesting rapid proteasomal degradation of the artificial fusion protein, possibly due to irregular folding. All three components of the fusion protein comprising HPV16 E6, HPV16 E7 and the C-terminal FLAG tag were detected suggesting full-length translation.

### Experimental Confirmation of 16L1/L2-E7 VLP Integrity

**Fig 2:**
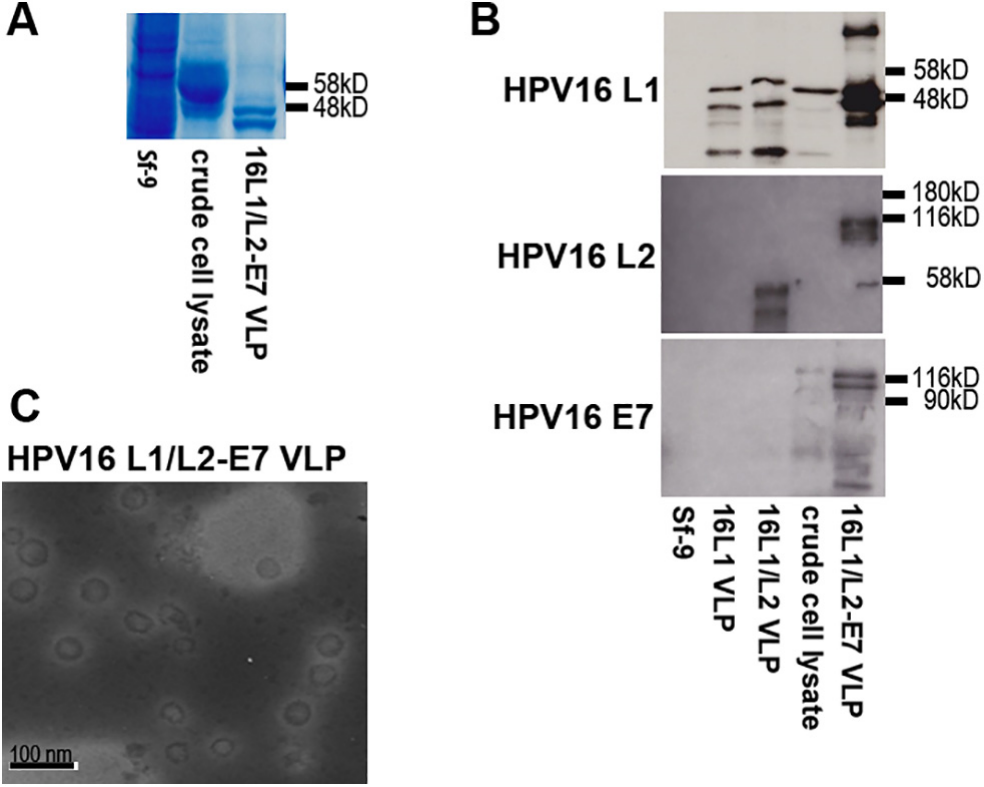
Generation of chimeric HPV16 L1/L2-E7 VLP. **A** Sf-9 insect cells were infected with recombinant HPV16 L1/L2-E7 baculovirus and VLP purified by sucrose cushion and CsCl gradient centrifugation. Purified VLP, infected, and uninfected Sf-9 cell lysates were analyzed by SDS-PAGE and Coomassie staining. **B** Antigenicity of HPV16 L1/L2-E7 VLP was assessed by Western blot using antibodies to HPV16 L1, HPV16 L2, or HPV16 E7. Purified HPV16 L1 VLP and HPV16 L1/L2 VLP were used as controls. **C** HPV16 L1/L2-E7 VLP were negatively stained by uranyl acetate and visualized by TEM at 30,000 fold magnification.

Immunization with HPV16 L1/L2-E7 VLP has been described to elicit an anti-TC-1 tumor response in mice, which was predominantly CTL-driven and perforin-dependent [25]. Therefore, we decided to use this system as standard for influenza A vaccine efficacy. Using recombinant baculovirus, HPV16 L1 and a fusion of HPV16 L2-E7 were co-expressed in Sf-9 insect cells, and co-assembled VLP purified by sucrose cushion and CsCl density gradient centrifugation. Analysis of infected crude Sf-9 cell lysate and purified VLP by SDS-PAGE and Coomassie staining revealed a prominent 50–55 kD band corresponding to viral L1 protein (Fig 2A), and a characteristic double band corresponding to purified L1 VLPs (Fig 2A). Western blot screening for L1 revealed a double band for purified L1/L2-E7 VLPs and a single 55 kD band for L1/L2-E7 in crude lysate (Fig 2B). HPV16 L1 VLPs and HPV16 L1/L2 VLPs (Fig 2B) served as controls. HPV16 L2 immuno-reactivity was shown for purified HPV16 L1/L2-E7 VLPs, with the L2-E7 fusion-protein migrating at approximately 116 kD (Fig 2B), and HPV16 L1/L2 VLPs [with wt L2] migrating around 60 kD (Fig 2B). An explanation for negative L2 staining observed for crude cell lysate may be the proportionally lower concentration of L2. HPV16 E7 antiserum reacted with purified 16L1/L2-E7 VLPs and slightly with crude cell lysate (Fig 2B), suggesting efficient co-assembly of HPV16 L1 with the HPV16 L2-E7 fusion into VLPs. Finally, transmission electron microscopy (TEM) used to assess VLP morphology revealed high numbers of correctly formed spherical viral capsids of ~ 50–60 nm in diameter (Fig 2C).

### Immunization of C57BL/6 Mice with Recombinant Influenza A Virus Induces IFN-γ-Producing HPV16 E6- and E7-Specific CD8^+^ T Cells

**Fig 3:**
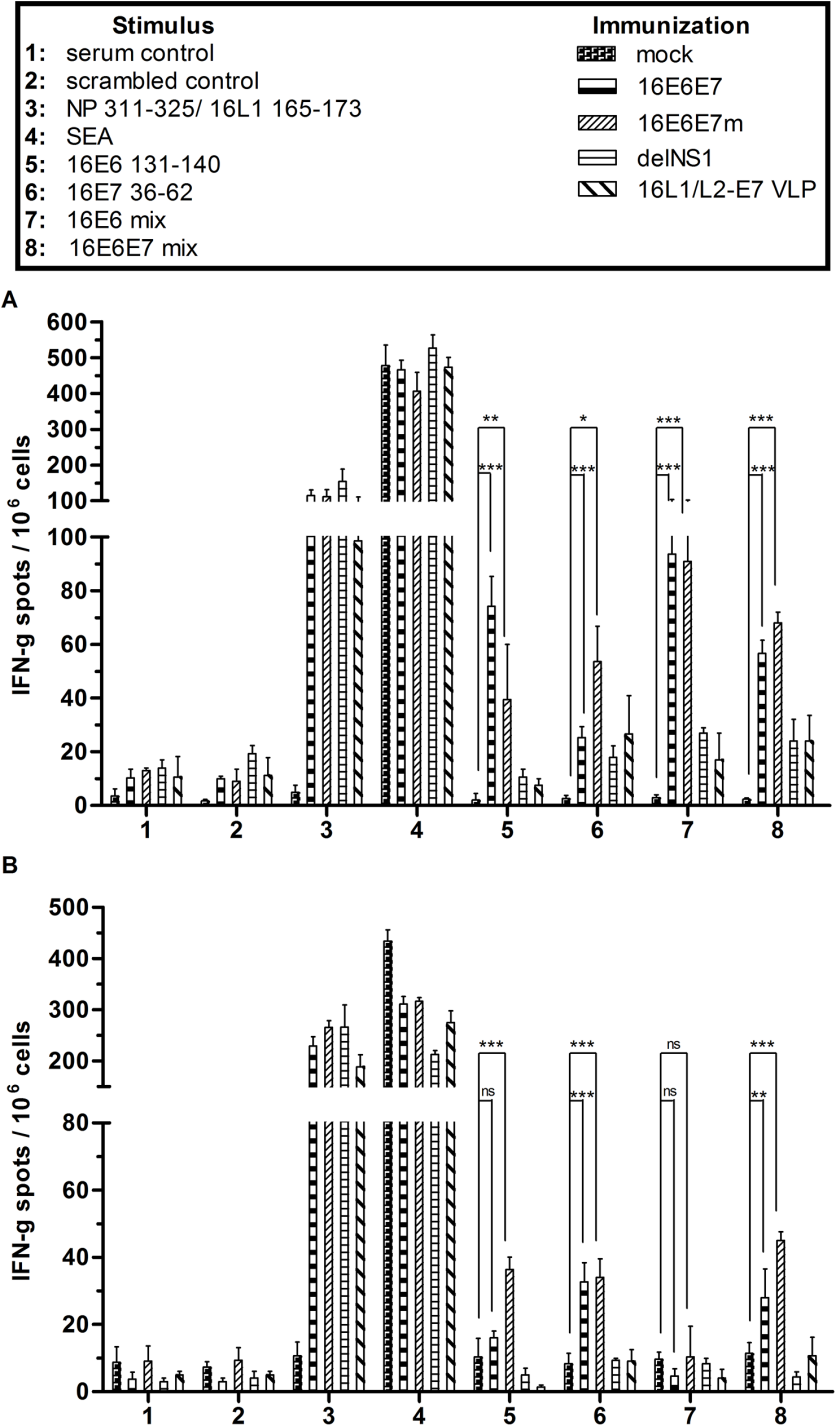
Recombinant 16E6E7 and 16E6E7m influenza viruses elicit HPV-specific CTL responses in mice. Four groups of mice (4 per group) were primed with PBS (mock), H1N1 16E6E7 virus, H1N1 16E6E7m virus, parental H1N1 delNS1 virus, or 16L1/L2-E7 VLPs, and boosted 10 days later with PBS, corresponding H3N2 serotypes, or VLPs. Mice were sacrificed **A** 10 days or **B** 30 days after boosting, splenocytes were isolated and stimulated in triplicates for 24 h with antigen peptides, SEA or medium alone. For mock and influenza A virus-vaccinated animals, NP_311-325_ peptide, for mice immunized with 16L1/L2-E7 VLP, 16L1_165-173_ peptide were used as positive controls. Shown are numbers of IFN-γ spots, counted under a light microscope, and plotted as mean ± SD of triplicate wells. One representative experiment of two is shown. Statistically significant differences for 16E6E7 or 16E6E7m compared to mock are indicated as asterisks (*** p<0.001, ** p<0.01, * p<0.05, ns not significant).

C57BL/6 mice (4 animals per group) were primed with recombinant H1N1 16E6E7 or 16E6E7m virus, parental H1N1 delNS1 virus, HPV16 L1/L2-E7 VLPs, or PBS as a control and boosted 10 days later with corresponding H3N2 viruses, VLPs, or PBS (Fig 3). The recombinant influenza and the VLP subunit vaccines showed no side effects and were as well tolerated as the PBS mock vaccination. To characterize the elicited cellular immune response, mice were sacrificed 10 or 30 days after the boost. Then splenocytes were isolated, stimulated with peptides harboring known CTL epitopes of HPV16 E6, HPV16 E7, or combinations thereof, and specific IFN-γ production was analyzed by ELISpot. As controls, SEA or peptides harboring known CTL epitopes of influenza A NP and HPV16 L1 were used for stimulation, respectively. Splenocytes from mice vaccinated with influenza A vectors or HPV16 L1/L2-E7 VLPs produced high levels of IFN-γ when stimulated with NP or L1 peptide (Fig 3A, group 3). As expected, pronounced IFN-γ production was observed for all groups when using SEA as stimulus (Fig 3A, group 4). Only cells from mice treated with recombinant 16E6E7 or 16E6E7m influenza viruses and stimulated with HPV16 E6_131-140_ and HPV16 E7_36-62_ (Fig 3A, groups 5,6), but not with HPV16 E6_41-50_ or E6_91-100_ (S2 Fig), revealed a specific and statistically significant IFN-γ response as compared to PBS-treated mice. A mixture of all E6 and E7 peptides also induced increased IFN-γ production in mice immunized with recombinant influenza viruses (Fig 3A, groups 7, 8) as compared to mock control mice. Interestingly, animals vaccinated with HPV16 L1/L2-E7 VLPs revealed a CTL response against E7 peptides that was lower when compared to 16E6E7m influenza A-vaccinated animals (Fig 3A, group 6). However, IFN-γ production was observed upon stimulation with HPV16 L1_165-173_ peptide for animals vaccinated with HPV16 L1/L2-E7 VLPs, indicating a possible immune-dominance of the L1 capsid (Fig 3A, group 3).

To assess the persistence of specific cellular immunity, splenocytes were isolated from as above vaccinated animals 30 days after the boost and ELISpot experiments conducted as described (Fig 3B). Overall, a 2.5-fold decrease of specific CTL responses to HPV16 E6 and E7 peptides was noted (Fig 3B; groups 5, 6, 8) and no synergistic stimulation by combined E6 peptides was observed for mice vaccinated with either recombinant influenza virus (Fig 3B; group 7). Nonetheless, an E6-E7-specific and statistically significant IFN-γ response remained detectable in recombinant influenza A-vaccinated mice when compared to mock-immunized animals.

Furthermore, influenza vector-induced humoral immune responses against HPV16 E6 and E7 were addressed. To this aim, sera of immunized mice were isolated and subjected to ELISA, using HPV16 E6 or E7 peptides harboring previously described humoral epitopes, scrambled control peptides, whole parental H1N1 or H3N2 viruses, or HPV16 L1 VLPs as antigens. Antibodies to HPV16 E6 and E7 peptides could not be detected in sera of mice vaccinated with recombinant 16E6E7 and 16E6E7m viruses or VLPs. In contrast, antibodies directed against influenza A or HPV16 VLPs were detected in sera from mice vaccinated with recombinant or parental influenza A viruses, or HPV16 L1/L2-E7 VLPs (S3 Fig). These results are suggestive for humoral immunodominance of influenza A virus, whilst the transgenic E6E7 fusion protein presented in an endogenous cytokine environment triggers cell-mediated immunity. The observed rapid degradation of the fusion protein may enhance this effect. In addition, the L1 capsid protein was immunodominant over the L2-E7 fusion-protein present as minor component in the co-assembled VLPs.

### Mice Vaccinated with Recombinant 16E6E7 or 16E6E7m Influenza Viruses Are Protected Against or Less Susceptible to Challenge with TC-1 Tumor-Cells

**Fig 4:**
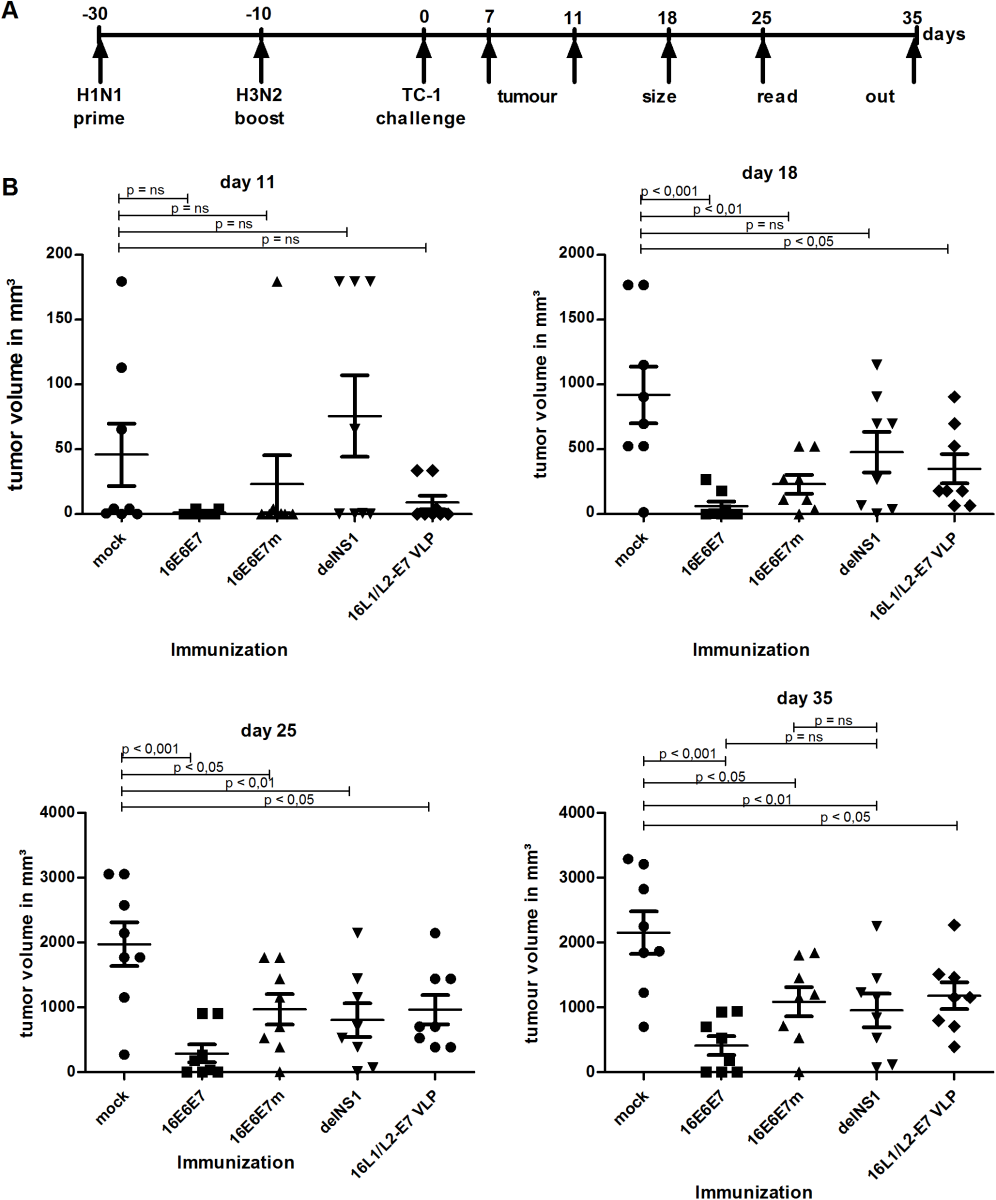
Mice s.c. vaccinated with recombinant 16E6E7 or 16E6E7m influenza viruses are partially protected from TC-1 induced tumors. **A** Schematic illustration of the experimental set up. **B** Groups (n = 8) of C57BL/6 female mice were vaccinated s.c. with PBS (mock), H1N1 16E6E7, H1N1 16E6E7m, parental virus (delNS1), or HPV16 L1/L2-E7 VLP, boosted 20 days later either with PBS, the corresponding H3N2 influenza serotype, or VLPs and challenged s.c. with 5_x_10^4^ TC-1 cells 10 days later. Animals were monitored once per week. Mean tumor volumes ± SEM of individual animals of one representative experiment of two are shown. Statistical significances of differences recorded for immunized groups and mock treated animals, or immunized groups and parental virus treated animals, were calculated by 1-way ANOVA and subsequent Dunnett’s multiple comparison test. Days after TC-1 tumor inoculation are indicated.

Given the E6-E7-specific cell mediated immune response generated by recombinant influenza A immunization, we analyzed whether vaccination could protect animals from challenge with HPV16 E6- and E7-expressing TC-1 tumor cells [24]. Groups of C57BL/6 mice (8 per group) were PBS–injected, or vaccinated with recombinant H1N1 16E6E7, 16E6E7m, parental viruses, or HPV16 L1/L2-E7 VLPs. Following booster immunization with PBS, respective H3N2 serotypes, or HPV16 L1/L2-E7 VLPs on day 20, mice were challenged by s.c. inoculation with TC-1 cells into the right flank, and tumor growth was monitored over 35 days (Fig 4A). From day 11 to day 18 tumor growth was most pronounced in animals injected with PBS or parental virus (delNS1). On day 18, all mock- and parental virus-immunized animals had developed palpable tumors, with volumes ranging between 14 and 1,700 mm³ for mock-treated animals and 33 and 1,150 mm³ for parental virus-treated mice, whereas 4 of 8 animals vaccinated with 16E6E7 influenza virus were clinically tumor-free (Fig 4B, day 18). In mice treated with 16E6E7m influenza virus, the mean tumor volumes were significantly lower (p < 0.01) than in mock-treated animals. Mice immunized with 16E6E7 viruses were significantly (p < 0.001) protected when compared to mock-treated animals (Fig 4B, days 25 and 35). Similarly, significant protection (p < 0.05) was observed for 16E6E7m-immunized mice albeit to a lesser extent (Fig 4B, days 25 and 35). Overall, 25% of mice vaccinated with recombinant influenza A viruses were completely protected from TC-1 tumor cell challenge. When compared to parental influenza virus, no statistical significance was observed for 16E6E7 and 16E6E7m recombinant influenza viruses (Fig 4, day 35). The observed results are likely due to an unspecific, influenza vaccine-induced innate immune response. In mice immunized with HPV16 L1/L2-E7 VLP, tumor cell growth was initially delayed (Fig 4B, day 11) and mice developed significantly smaller lesions when compared to PBS-injected mice. In comparison to 16E6E7-vaccinated animals, mice were less protected against tumor development when immunized with 16E6E7m, parental influenza virus, or 16L1/L2-E7 VLP (Fig 4B, lower panels), indicating a possible advantage of the live 16E6E7 influenza vaccine approach over subunit VLP vaccination.

### Subcutaneous Immunization of C57BL/6 Mice With Recombinant 16E6E7 or 16E6E7m Influenza Viruses Delays Growth of Established TC-1 Tumors

**Fig 5:**
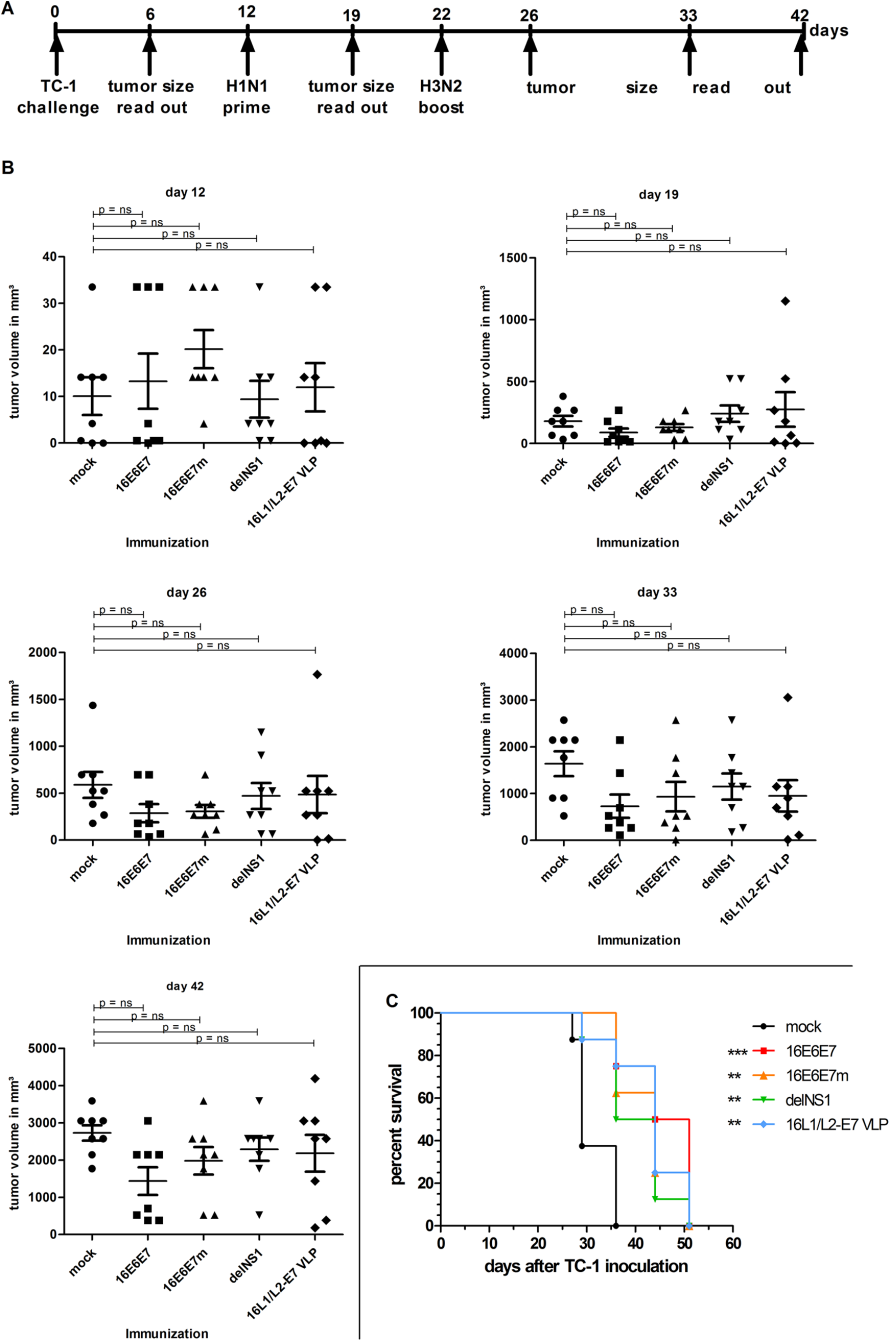
Subcutaneous vaccination with recombinant 16E6E7 or 16E6E7m influenza viruses delays growth of established TC-1 tumors and prolongs survival of tumor bearing mice. **A** Schematic illustration of the experimental set up. **B** Groups (n = 8) of C57BL/6 female mice were challenged with 5_x_10^4^ TC-1 cells on day 0 and tumor growth monitored. Mice were primed s.c. after palpable tumors had developed on day 12 with PBS (mock), H1N1 16E6E7, H1N1 16E6E7m, parental influenza virus (delNS1), or HPV16 L1/L2-E7 VLP, and boosted 10 days later as described. Animals were monitored once per week. Mean tumor volumes ± SEM of individual animals of one representative experiment of two are shown. Statistical significance of differences recorded for immunized groups and mock treated animals were calculated by 1-way ANOVA and subsequent Dunnett’s multiple comparison test. Days after TC-1 tumor inoculation are indicated. **C** Termination criteria comprised rapid weight loss, tumor diameters >16 mm, tumor ulceration, scrappy fur and diarrhea. Days indicate time after TC-1 tumor cell inoculation. One representative experiment of two is shown. Asterisks indicate p-values compared to mock (*** p<0.001, ** p<0.01).

To address the therapeutic potential of recombinant 16E6E7 and 16E6E7m influenza viruses, 40 mice were inoculated with TC-1 cells on day 0 and monitored for tumor development. When ≥50% of animals had developed palpable tumors (day 12; Fig 5B,), mice were randomly assigned to 5 groups (8 animals/group), and subcutaneously injected with PBS (mock control), recombinant 16E6E7, 16E6E7m, parental viruses, or HPV16 L1/L2-E7 VLP, and boosted ten days later (Fig 5A).

Nineteen days post inoculation (Fig 5B) mean tumor volumes amounted to 179 mm³ in control animals, 65 mm³ in 16E6E7-treated animals, and 113 mm³ in 16E6E7m-injected mice. At day 26, mean tumor volumes appeared to be slightly reduced in 16E6E7- and 16E6E7m-vaccinated groups (Fig 5B, day 26) in comparison to mock and parental virus control groups. This trend persisted until day 33 and was most pronounced in 16E6E7-vaccinated followed by 16E6E7m-immunized mice (Fig 5B), yet not statistically significant in comparison to mock controls. From day 33 to day 42, tumor sizes increased rapidly in all groups, which is suggestive for a transient vaccination-induced immune response (Fig 5B, day 42).

Then, survival rates of vaccinated mice versus mock controls were analyzed by Kaplan–Meier test (Fig 5C). Mice were prematurely sacrificed according to pre-defined termination criteria such as rapid loss of body weight (>50%), tumor diameters >16 mm, scrappy fur, diarrhea, and/or tumor ulceration. According to these criteria, 1 mock treated mouse was euthanized on day 27 post TC-1 inoculation. After 29 days, more than 50% of control mice had to be sacrificed, whilst 100% of 16E6E7- and 16E6E7m-treated animals remained alive. After 36 days, 100% of mock treated animals and 50% of mice vaccinated with parental influenza virus fulfilled premature termination criteria, whilst 75% of 16E6E7 and 62.5% of 16E6E7m treated animals did not. On day 44, survival of 16E6E7m-immunized animals dropped to 25%, whilst 50% of 16E6E7 vaccinated animals were still in good condition. On day 44, 12.5% of parental virus-treated mice and 50% of VLP-immunized animals could be kept alive. On day 51, all remaining mice were euthanized according to termination criteria. Observed survival rates were significantly higher in all vaccine groups than in mock controls, as shown by a Mantel-Cox-Test (Fig 5C).

### Intratumoral (i.t.) Application of Recombinant 16E6E7 and 16E6E7m Influenza Virus Reduces Growth of or Eradicates Established TC-1 Tumors in Mice

**Fig 6:**
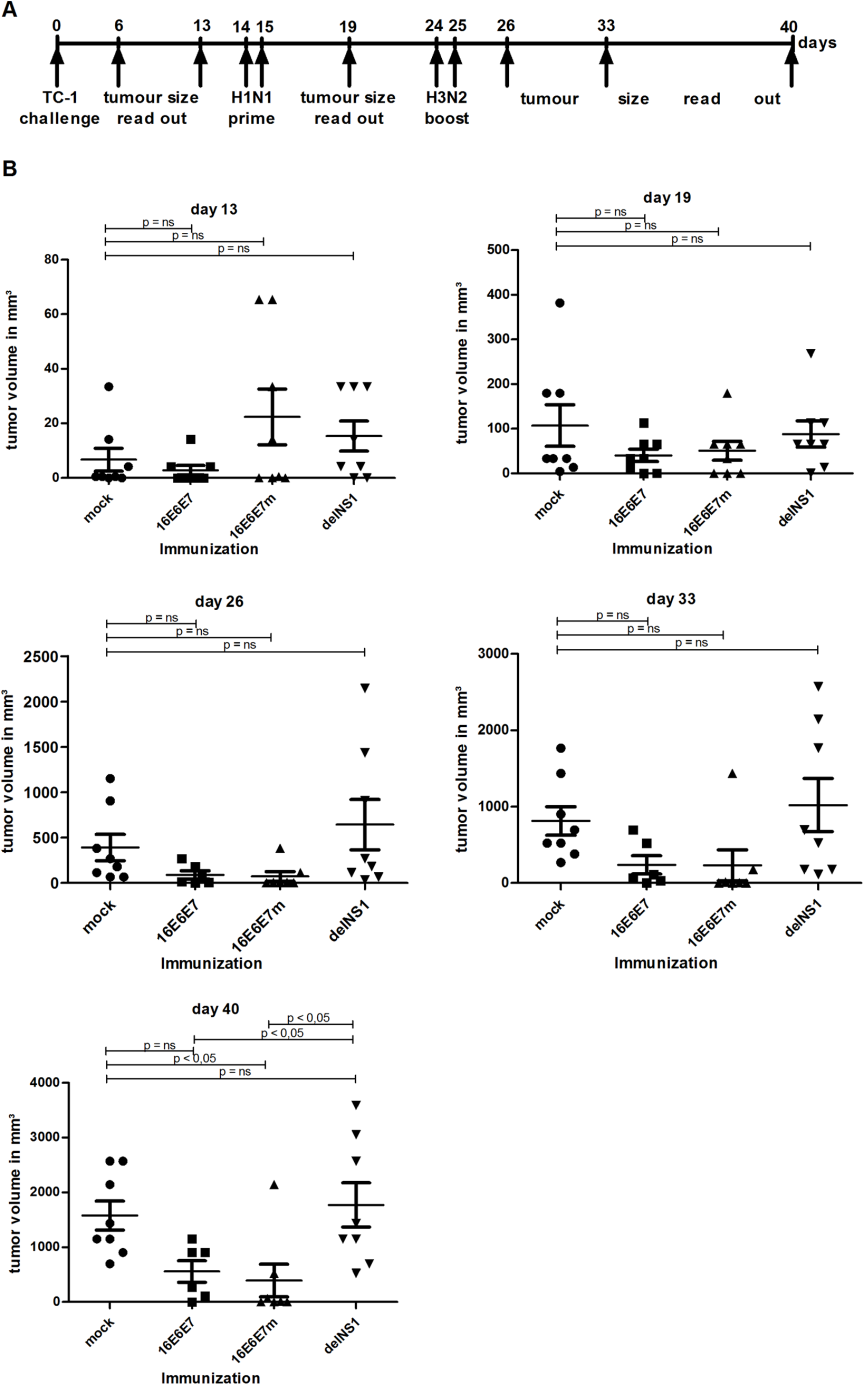
Intratumoral (i.t.) vaccination with recombinant 16E6E7 and 16E6E7m influenza viruses delays growth of, or eradicates established TC-1 tumors. **A** Schematic illustration of the experimental set up **B** Five groups of female C57BL/6 mice (8 per group) were inoculated with 5x10^4^ TC-1 cells on day 0 and tumor growth monitored. After palpable tumors had developed, mice were primed i.t. on day 13 and 14 with PBS (mock), H1N1 16E6E7, H1N1 16E6E7m, parental influenza virus (delNS1), or HPV16 L1/L2-E7 VLP, and boosted either with PBS, the corresponding H3N2 influenza serotype, or VLPs 10 days later. Animals were monitored once per week and mean tumor volumes ± SEM of individual animals of one representative experiment of two are shown. Statistical significance of differences recorded for immunized groups and mock treated animals, or immunized groups and parental virus treated animals, were calculated by 1-way ANOVA and subsequent Dunnett’s multiple comparison test. Days after TC-1 tumor inoculation are indicated.

In a next step, the therapeutic potential of intratumoral vaccine administration was assessed. To this aim, four groups (8 animals/group) of C57BL/6 mice were inoculated with 5_x_10^4^ TC-1 tumor cells and monitored for tumor development (Fig 6A). After 14 days, when at least 50% of animals of each group had developed palpable tumors, mice were i.t. injected with recombinant 16E6E7, 16E6E7m, parental influenza virus, or PBS and then boosted ten days later, which led to the loss of three animals (two 16E6E7-, one 16E6E7m-injected animals). A possible explanation for this immediate fatal outcome may be vascular shock since TC-1 tumors were well established and heavily vascularized and high injection volumes might be less tolerable under such conditions. However, all other animals did not show any side effects during and after vaccinations.

A 40-days follow-up revealed delayed tumor growth in mice immunized i.t. with recombinant influenza viruses when compared to parental virus and PBS control groups: on day 40, the mean tumor volume was significantly (p < 0.05) lower in 16E6E7m vaccinated mice (Fig 6B, day 40). A likewise, yet weaker effect was also achieved by E6E7-treatment. Strikingly, 25% of i.t. influenza vaccine-treated mice (1 animal of the E6E7- and 3 animals of the E6E7m-treated groups) showed complete tumor regression, and no recurrence was noted within the observation period (70 days; not shown). Consistent with in vivo vaccine efficacy, peptide-based ELISpot experiments revealed antigen-specific IFN-γ production by splenocytes of mice vaccinated i.t. with recombinant 16E6E7 or 16E6E7m influenza A viruses, whilst parental virus and PBS controls scored negative in this assay (S4 Fig).

## Discussion

Therapeutic HPV vaccination strategies with proven efficacy are still lacking. HPV induced neoplasias invariably retain and express the viral E6 and E7 genes, which argues for the use of these non-self viral oncoproteins as therapeutic immune targets thus circumventing the danger of inducing autoimmunity. Several animal studies and pre-clinical trials substantiate the power of this therapeutic concept [8,26]. DNA- or protein-based therapeutic HPV vaccines have been less successful, probably because antigen-presenting cells (APC) are not targeted and/or cytotoxic immunity against target antigens is not sufficiently induced. Thus, live-vector vaccines such as recombinant influenza A viruses that express the viral oncoproteins E6 and E7 are interesting alternative options. Considering patient safety in future clinical trials, deletion-inactivated HPV16 E6 and E7 genes were generated (16E6E7m) in addition to unmodified genes. Immuno-detection of HPV16 E6, E7, and the FLAG tag located at the C-terminus of the E6E7 fusion gene confirmed complete transgene translation and antigenicity. Interestingly, E6E7 fusion proteins were only detected in infected cells when the proteasomal machinery was inhibited, whereas mRNA expression was readily observed, suggesting protein instability and rapid intracellular degradation, presumably due to improper folding. Thus it appears that neither wt nor mutated HPV16 E6-E7 fusion proteins are able to persist and exert potentially oncogenic biological activity, and thus may be relatively safe in human application. Fusion protein degradation did not negatively affect induction of a cell-mediated immune response. This is consistent with the assumption that antigen degradation constitutes a pre-requisite for efficient T cell priming by e.g. dendritic cells (DCs). One principle drawback of viral vector vaccines is the induction of neutralizing antibodies to the vector that may abrogate efficiency of booster immunizations. Live-attenuated influenza A offers a variety of serotypes that can be employed for prime and boost vaccination to circumvent this potential problem.

Intranasal immunization with recombinant influenza viruses has potential advantages such as ease of application, stimulation of mucosal and systemic immune responses with induction of secretory IgA and cell-mediated immunity, and obviates the need for injection. However, in practice immune responses to the transgenes may be more difficult to achieve, and vaccine antigens may be unstable or readily cleared from mucosal sites. In contrast by s.c. or intralesional administration route, one major immune barrier i.e. mucosal surface with possibly pre-existing strong local immunity from natural infection is avoided.

Most target populations for recombinant E6E7/E6E7m influenza vaccination are expected to have pre-existing immunity to influenza virus, which is expected to confer type-restricted reduction in vaccine efficacy. However, studies indicate that protective antibody responses induced in children and young adults last approximately for 6 months with titers decreasing considerably within this time frame [27]. Since anti-influenza antibody titers are lower in adults and elder, and the probability of HPV-related disease increases with age, seasonal influenza vaccination might be less likely to negatively impact E6E7/E6E7m influenza vaccine efficacy. Nevertheless, pre-vaccination serology and the use of HA subtypes not circulating in the population are strategies to increase the effectiveness of recombinant E6E7/E6E7m influenza vaccination in humans.

Live-attenuated influenza A viruses have been shown to induce a strong cell-mediated immune response [14], which may be further enhanced by the use of suitable adjuvants. Incomplete deletion of the NS1 ORF leads to type I IFN sensitivity and thereby attenuation of influenza A viruses, as the remaining N-terminal 11 aa of NS1 are non-functional in the expressed NS1-E6E7/E6E7m fusion proteins [28]. No difference in pathogenicity of recombinant live-inactivated influenza virus compared to parental virus was measurable in vitro. All viruses replicated comparably when cultivated in IFN-deficient Vero cells and reached similar titers, whereas replication was abolished when using IFN-competent cells (UACC62 or TC-1 cells) for viral passaging (not shown). Previously, attenuated delNS1 influenza A vectors have been used to express either lymphocyte-stimulating cytokines such as IL-2 as immune response enhancers, or the bacterial antigen ESAT-6 to induce protective immunity against tuberculosis [13,22]. As important safety feature, influenza viruses are RNA viruses that replicate without DNA intermediates. This excludes the possibility of genetic recombination events and dissipates the theoretical concern of E6-E7 oncogene integration into the host genome.

To eradicate (pre-) cancerous HPV-induced lesions by therapeutic vaccination, induction of a robust cell mediated immune response is considered necessary. Many approaches like DNA- or protein-based vaccines require strong adjuvants to direct the immune system towards a T_H1_ response [29,30]. In contrast, attenuated influenza A virus can serve as its own “adjuvant” to generate robust cellular immunity [31]. Infected cells attempt to produce type I IFN to obtain an antiviral state that blocks viral replication, and to create a cytokine environment that promotes a cell mediated immune response against virus-infected cells. The viral protein NS1 compromises this cellular reaction by antagonizing type I IFN. By partial deletion of NS1 and insertion of HPV16 E6 and E7 genes into the NS1 deletion site, recombinant delNS1 influenza A viruses cannot escape from immune surveillance and thus become replication-deficient as shown in mice and humans [20]. Nonetheless, infected cells can express and present epitopes of HPV16 E6 and E7 in an environment that promotes T_H1_ mediated immunity.

In this work, IFN-γ production served as read-out for this type of immune response. In vivo and ELISpot experiments have demonstrated that recombinant influenza A viruses induced HPV16 E6- and E7-specific CTL that produced IFN-γ in response to stimulation with short E6 and E7 peptides for five weeks, irrespective of the vaccine administration route (s.c. or i.t.). In contrast, animals vaccinated with recombinant VLPs exerted significantly less potent IFN-γ responses upon stimulation with E6 and E7 peptides as compared to recombinant influenza-vaccinated mice. As possible explanation, the immune responses against VLPs may be primarily of T_H2_ type, contrary to T_H1_-driven immunity induced by recombinant influenza viruses.

Generally, induction of a humoral immune response by therapeutic vaccination is not desired as it may have unwanted adverse effects. A cell-mediated immune response against target antigens E6 and E7, but no HPV16 E6- or E7-reactive antibodies were detected in sera from recombinant influenza virus-immunized mice. Sera contained antibodies directed against H1N1 or H3N2 influenza virus strains, thus underlining the advantage of using different influenza vaccine strains for prime and boost immunizations.

Recombinant influenza A viruses were designed for therapeutic vaccination against HPV-induced disease and thus to induce primarily a cell-mediated immune response. Nevertheless, the prophylactic efficacy of recombinant influenza virus was also evaluated in vivo, using a well-established murine TC-1 tumor model. Interestingly, tumor cell challenge of 16E6E7 influenza virus-vaccinated mice resulted in significantly lower mean tumor volumes when compared to mock-treated mice (p < 0.001), or in complete protection against tumor development (50% of animals). In contrast, animals immunized with 16E6E7m virus could not sustain protection: 7 of 8 animals had developed palpable tumors 35 days after TC-1 challenge. Overall, tumor growth was significantly reduced in all animals vaccinated with recombinant virus prior to challenge. Interestingly, mice vaccinated with parental virus were also significantly protected when compared to PBS-immunized control animals, although no evidence for a cell mediated E6-E7-specific immune response was found and all animals had developed tumors at day 35. The biological mechanisms underlying oncogene-independent rejection might be attributed to the induction of non-specific cytotoxic immunity [31] and/or enhanced tumor surveillance via non-specific stimulation of the innate immune system by influenza A virus infection. Contrary to licensed VLP-based prophylactic vaccines which are effective only when given to naïve (uninfected) individuals, recombinant influenza viruses expressing E6 and E7 were designed to induce a therapeutic effect against pre-existing infections or lesions. The sterilizing humoral immune response induced by licensed L1 VLP-based vaccines neutralizes virions prior to infection of target cells. In contrast, the viral oncoproteins E6 and E7 are expressed by infected cells and targeted by a cellular immune response induced by recombinant E6E7/E6E7m influenza viruses. Animals immunized with recombinant or parental influenza viruses showed significantly prolonged survival but eventually succumbed to tumor disease. This result suggests that vaccinations of mice induce a cell-mediated immune response, which, however, does not persist, or is circumvented over time by tumor escape mechanisms. Therefore, the T_H1_ mediated immune response needs to be enhanced in order to improve the therapeutic efficacy of the recombinant influenza vaccine. For application in humans, influenza A viruses expressing cytokines that stimulate the adaptive immune system or T_H1_ type immune responses could be designed. Previous studies on a DNA-based therapeutic HPV vaccine have shown that boost injection with DCs pulsed with the HPV16 E7 epitope RAHYNIVTF can induce a strong cell-mediated immune response against TC-1 tumor cells in mice [32]. A similar effect may be achieved by combined administration of epitope-pulsed DCs and recombinant influenza A viruses. Further options include the inactivation of HPV16 E6- and E7-specific tolerogenic immune cells such as CD25^+^ FoxP3^+^ regulatory T cells, or the addition of the universal T cell epitope PADRE to the HPV16 E6E7 fusion protein to enhance T helper cell proliferation.

Intratumoral administration of 16E6E7 or 16E6E7m viruses led to complete regression of TC-1 tumors in several mice (25%) and delayed tumor progression in the remaining animals (Fig 6B). Recombinant influenza viruses were likely able to infect TC-1 tumor cells in vivo, yet could not replicate productively due to the presence of type I IFN and the lack of trypsin needed for HA activation. This would explain the observed tumor regression, as infection would have induced type I IFN and, consequently, abrogation of immune evasion by tumor cells. We may speculate that replication-competent recombinant influenza viruses can further enhance anti-tumor immunity. The ability of recombinant influenza A viruses to infect TC-1 cells was indeed confirmed in vitro by detection of viral NP and E6E7 mRNA (S5 Fig). However, recombinant influenza A viruses were unable to replicate in, or lyse TC-1 cells. Therefore i.t. administration of recombinant influenza A viruses offers the advantage of guiding T cells directly to the tumour site and inducing two-fold anti-tumoral immune-responses. Tumor cells are targeted by immune cells specific for HPV16 E6 and E7 and also for influenza. These mechanisms likely increase vaccine efficacy and therefore circumvent possible shortcomings of the 16E6E7m protein compared to 16E6E7 wt protein. Induction of type I IFN further enhances this immune response. In contrast these potential advantages were not utilized by s.c. application route. Furthermore live-attenuated influenza viruses can exert oncolytic activity, and antigen-specific CTL [33] and natural killer cells are recruited via lysis of tumor cells [34]. Oncolytic efficacy could be further enhanced by co-administration of recombinant influenza viruses expressing immune-stimulatory cytokines such as IL-2 [22] or IL-15 [35]. Also modification of viral HA molecules that allows activation by proteases present in the tumor, other than trypsin used in this work, might enhance replication of recombinant influenza viruses and their oncolytic capacity. HPV-induced anogenital high-grade squamous intraepithelial lesions (HSIL) often arise at multiple (multifocal) sites and may require aggressive, possibly disfiguring surgery. Intralesional administration of recombinant 16E6E7 influenza virus may be envisaged as tissue-preserving alternative to surgical ablation of HSIL. Tumor escape mechanisms such as E7-mediated induction of indoleamine-2,3-oxygenase, which antagonizes IFN-γ [10], need to be further elucidated to efficiently circumvent tumor immune evasion strategies.

In summary, it is shown that recombinant influenza A HPV16-E6E7 viruses have the potential to promote regression of HPV16-associated tumors via induction of a cellular immune response. Studies aiming at further optimizing the efficacy of this therapeutic approach are ongoing.

## Materials & Methods

### Cells

The murine TC-1 tumor cell line, generated through stable transfection of C57BL/6 lung cells with HPV16 E6, E7 and oncogenic *ras* as described [24], was a kind gift from T.C. Wu (Johns Hopkins, Baltimore) and was maintained in RPMI 1640 supplemented with 10% FCS, penicillin, streptomycin and 0.4 mg/ml G418. The type I IFN deficient Vero cell line derived from kidney epithelial cells of an African green monkey [36], was used for influenza A virus propagation and cultivated in serum-free Opti-Pro medium supplemented with penicillin, streptomycin and L-glutamine. All mammalian cell lines were cultivated at 37°C, 5% CO_2_ and 95% humidity. *Spodoptera frugiperda* (Sf9) insect cells were cultivated in Grace’s medium supplemented with 5% FCS and 0.5% Pluronic at 27°C. All cell culture media and supplements were obtained from Gibco Life Technologies.

### Generation of the HPV16 E6E7 and E6E7m Constructs

An HPV16 E6E7 fusion gene consisting of wt E6 and E7 ORFs and a FLAG tag (FL) was designed and synthesized (GeneArt, Life Technologies). For safety considerations a similar fusion construct with mutated (mut) E6 and E7 genes was also generated (HPV16 E6E7m). To this aim, codons encoding HPV16 E6 amino acids (aa) 73–77 and 146–151, and HPV16 E7 aa 21–24 reported to assure biological activity of these oncoproteins [5,37,38], were deleted. The synthetic fusion genes were sub-cloned into a pHW2000 plasmid derivative [39] containing a short NS1 segment truncated to 11 N-terminal aa (delNS1) and under the control of a Pol I and Pol II promoter for vRNA and mRNA expression, resulting in a delNS1 vector encoding HPV16 E6, E7 (both either wt or mut) and FL (pHW-delNS1-16E6E7FL and pHW-delNS1-16E6E7mFL). To enhance transgene expression, the splice donor site was mutagenized according to [40], and pHW-delNS1-16E6E7FL or pHW-delNS1-16E6E7mFL plasmids were used as templates for PCR-directed mutagenesis, using primers: (forward) 5'-TGT GTC AAG CTT TCA GGT ATT TGC CGC AAT-3', (reverse) 5’-CTG TGT TAT CAT TCC ATT CAA GTC C-3’ (VBC Biotech) and Phusion™ proofreading polymerase (Finnzymes). Amplicons were analyzed by agarose gel electrophoresis, gel-extracted by Qiaquick kit (Qiagen) and blunt-end cloned into pCR2.1 by TOPO cloning procedure (Invitrogen), resulting in constructs pCR2.1-16E6E7FLsp and pCR2.1-16E6E7mFLsp. These were *Xcm I* (New England Biolabs) and *Hind III* (Roche) digested. Gel-purified digestion products were re-ligated using T4 DNA ligase (Roche). Resulting constructs pHW-delNS1-16E6E7FLsp and pHW-delNS1-16E6E7mFLsp were used for subsequent generation of recombinant influenza viruses.

### Generation of Recombinant Influenza A Viruses

Four recombinant influenza A viruses were obtained as previously described [35]: H1N1 delNS1 16E6E7FL (H1N1 16E6E7), H1N1 delNS1 16E6E7mFL (H1N1 16E6E7m), H3N2 delNS1 16E6E7FL (H3N2 16E6E7) and H3N2 delNS1 16E6E7mFL (H3N2 16E6E7m). The internal segments have been derived from the IVR-116 vaccine strain (WHO) that originated from A/Puerto Rico/8/34 for PA, PB2, NP, M, and A/Texas/1/77 for PB1. The viral HA and NA genes were derived from H1N1 virus A/New Caledonia/20/99 and H3N2 virus A/Aichi/2/68 for. Ninety percent confluent Vero cells were co-transfected with 0.5 μg of plasmid pHW-delNS1-16E6E7FLsp or pHW-delNS1-16E6E7mFLsp, expressing both vRNA and mRNA, and 0.5 μg of each bidirectional plasmid encoding the remaining seven segments using Nucleofection technique (Amaxa). Recombinant influenza A viruses and parental H1N1 delNS1 and H3N2 delNS1 viruses were collected with culture supernatant after cultivation for 4–5 days.

### Verification of Transgene Stability and Expression

To verify transgene stability (in the delNS1 reading frame), recombinant influenza viruses were passaged in Vero cells for at least five rounds. Twenty four hours post infection, viral RNA was isolated using the QIAamp viral RNA mini kit (Qiagen) and subjected to RT-PCR using the uni-12 primer: 5’-AGC AAA AGC AGG-3’ (VBC Biotech) for cDNA generation. To amplify the wt and mutated NS1-16E6E7 fusion genes from recombinant viruses, the following PCR primers were used: (forward) 5´-AGC AAA AGC AGG GTG ACA AAG-3’, (reverse) 5´-CTC TTG CTC CAC TTC AAG C-3’.

To verify transgene mRNA expression, Vero cells were grown to sub-confluence in 6 well plates, infected with recombinant influenza A viruses (10^5^ pfu) and incubated for 12 hours. Cells were lysed using TRI-Reagent (Sigma Aldrich) and total RNA was extracted via phase separation using chloroform, followed by isopropanol precipitation and re-suspension of RNA in RNase free water. Subsequent reverse transcription was carried out using a first strand cDNA synthesis kit (Roche) and mRNA-specific oligo dT primers. Obtained cDNA served as template for PCR amplification of the HPV 16 E6E7 fusion using the following primers: (forward) 5’-ATG TTT CAG GAC CCA CAG GAG CGA-3‘, (reverse) 5’-TTT ATC GTC ATC GTC CTT GTA GTC-3‘ (VBC Biotech).

For protein analysis, Vero cells were grown to sub-confluence in the presence or absence of 10 μM MG-132 dissolved in DMSO for 12 hours, infected with recombinant influenza A viruses (10^5^ pfu) and then incubated for 12 hours in the presence of MG-132. Cells were harvested, lysed in Laemmli-sample buffer, boiled for 5 min and analysed by SDS-PAGE and Western blot.

### Production of High-Titer Recombinant Influenza A Viruses

Vero cells were seeded at a density of 3_x_10^4^ cells/cm² in 125 cm² tissue culture flasks, cultivated for 24h and infected with recombinant virus at multiplicity of infection (MOI) of 0.001 in Opti Pro supplemented with 20 mM L-glutamine, 5 μg/ml trypsin and 0.5 μg/ml amphotericin B (all Sigma) in a total volume of 5 ml at 37°C for 1.5h. Culture medium was added to a final volume of 25 ml and infected cells cultivated for 48h observing cell lysis after 24 h. Virus titers were determined for Vero cells in virus growth medium containing Opti-Pro, 20 mM L-glutamine, 5 μg/ml trypsin and 0.5 μg/ml Amphotericin B (Sigma) by endpoint dilution assay. Vero cells were seeded at a density of 1.5_x_10^4^ cells/well the day before infection, viruses were diluted from 10^−3^ to 10^−9^ in virus growth medium, then cells were infected. Three days post infection cells were analyzed for cytopathic effects by confocal light microscopy. Virus titers were calculated as 50% tissue culture infectious dose per ml (TCID_50_/ml) according to the Reed and Muench method [39].

### Generation of HPV16 L1/L2-E7 Chimeric Recombinant Baculovirus and Production of Virus-Like Particles (VLP)

The double expression plasmid coding for the HPV16 L1 and the L2-E7 fusion was a kind gift of John T. Schiller (NIH). Recombinant baculovirus stocks were generated by co-transfection of Sf9 cells with baculovirus DNA (Baculo-Gold, BD Biosciences) using Lipofectin (Invitrogen), followed by plaque purification [41]. Sf9 cells were infected at a MOI of ~10 with recombinant HPV16 L1+L2-E7-expressing baculoviruses. After 72 hours, cells were lysed by boiling in SDS sample buffer and analyzed by SDS/PAGE and Coomassie staining or Western blotting. VLP were purified by sucrose/CsCl density gradient centrifugation [42], adsorbed to glow-discharged carbon-coated copper grids, fixed with 2.5% glutaraldehyde, negatively stained with 1% uranyl acetate and analyzed on a JEOL 1010 transmission electron microscope (TEM) at 80 kV with 30,000 fold magnification.

### Antibodies, SDS-PAGE and Western Blot

The antibodies used in these assays comprised monoclonal mouse anti(α)-influenza A NP blend clone A1/A3 (Millipore, 1:1,000 dilution); mouse α-Flag clone M2 (Sigma, 1:5,000); mouse α-HPV16 E7 clone ED17 (Santa Cruz, 1:200); mouse α-HPV16 E7 clone 8C9 (Invitrogen, 1:150); mouse α-HPV16 E6 clone 6F4 (Euromedex, 1:200); mouse α-HPV16 L1 Camvir-1 (BD Biosciences, 1:5,000); mouse α-pRb (1:500 dilution); polyclonal rabbit α-HPV16 L2 11–200 (described in [42], 1:5,000) and rabbit α-GAPDH (Santa Cruz, 1:1.000); goat α-rabbit HRP and goat α-mouse HRP (both from Biorad,1:25,000). Proteins were separated on 10 to 15% gradient Tris-glycine SDS gels (Biorad) using the Tris-glycine SDS buffer system. Western blotting was performed by electrophoretic transfer of proteins to a polyvinyl difluoride (PVDF) membrane (Millipore).

### Animals

Six to eight weeks old female C57BL/6 mice were obtained from Charles River Laboratories (Germany), and used for vaccination and challenge studies. Animals were kept under standard conditions at the Institute of Biomedical Research of the Medical University Vienna and provided with a standard diet and water ad libitum.

### Prophylactic Immunization Studies in Mice

On day 0, animals were randomly divided into 5 groups of 8. Groups 1, 2 and 3 were primed subcutaneously (s.c.) with 3.75 log TCID_50_ H1N1 16E6E7, 16E6E7m and parental H1N1 virus. Control groups 4 and five were s.c. injected with 125 μg HPV 16 L1/L2-E7 VLP and PBS (group 5). On day 20, mice were boosted s.c. with H3N2 16E6E7 (3.75 log TCID_50_) (group 1), H3N2 16E6E7m (group 2), parental H3N2 virus (group 3), 60 μg HPV16 L1/L2-E7 VLP (group 4), and PBS (group 5). Animals were challenged on day 30 with 5_x_10^4^ TC-1 cells administered s.c. into the right flank. Tumor growth was monitored once per week, diameters (d) were measured and volumes calculated according to the formula *V = 4/3*.*π*.*r* ^*3*^ *(r = d/2)*. Tumor size of mice that were euthanized before the study endpoint due to severe disease was calculated as tumor growth rate per day according to the formula *tumor growth rate/day = measured tumor diameter/day*.

### Therapeutic Subcutaneous (s.c.) Vaccination Studies in Mice

Animals were randomly divided into 5 groups of 8, s.c. challenged with 5_x_10^4^ TC-1 tumor cells into the right flank on day 0 and tumor development monitored twice per week. When 50% of animals of each group had developed palpable tumors, all mice were primed s.c. in the skin fold of the neck, with either H1N1 16E6E7 or 16E6E7m (groups 1 or 2; 3.75 log TCID_50_), corresponding wt H1N1 virus (group 3), 125 μg HPV16 L1/L2-E7 VLP (group 4), and PBS (group 5). Ten days after priming, animals were boosted with the corresponding H3N2 serotypes (groups 1–3), 60 μg VLP (group 4), and PBS (group 5). Tumor growth was monitored once per week and volumes calculated (*V = 4/3*.*π*.*r* ^*3*^ *)*.

### Therapeutic Intratumoral (i.t) Immunization Studies in Mice

Animals were randomly divided into 4 groups of 8, s.c. challenged with 5_x_10^4^ TC-1 tumor cells into the right flank on day 0 and tumor development monitored twice per week. As soon as 50% of animals of each group had developed palpable tumors, mice were primed with either H1N1 16E6E7 (group 1) or 16E6E7m (group 2), parental H1N1 virus (group 3), or PBS (group 4). Animals lacking palpable tumors at the day of priming were immunized s.c. at the site of tumor cell inoculation. Due volume constraints, mice received twice 250 μl (3.75 log TCID_50_ in 0.5 ml) of recombinant influenza viruses i.t. within two days, resulting in the desired total application volume of 500 μl/mouse. Ten days after priming animals of groups 1–3 were boosted with the corresponding H3N2 serotypes or PBS (group 4). Tumor growth was monitored once per week and tumor volumes calculated as described.

### Survival Studies

Mice were prematurely sacrificed by cervical dislocation, according to pre-defined termination criteria such as rapid loss of body weight (>50%), tumor diameters >16 mm, scrappy fur, diarrhea, and/or tumor ulceration and health of mice was monitored 3 times per week. No unexpected deaths of animals took place during survival studies and no analgesics had to be used.

### Assessing Cell-Mediated and Humoral Immunity

Animals were randomly divided into 5 groups of 4. On day 0, mice were primed s.c. or i.t. with H1N1 16E6E7 (group 1) (3.75 log TCID_50_), H1N1 16E6E7m (group 2), corresponding wt influenza A (group 3), 125 μg HPV16 L1/L2-E7 VLPs (group 4), or PBS (group 5). Mice were boosted s.c. or i.t. 10 days after priming with the corresponding H3N2 serotypes (groups 1–3), 60 μg HPV 16 L1/L2-E7 VLPs (group 4), or PBS (group 5). Following priming, animals were sacrificed by heart puncture under anesthesia (0.5 mg Rompun/ 3mg Ketamin per mouse) on day 20, or day 40 for long term assessments, and serum and spleens collected for ELISpot and ELISA experiments.

The E6-E7-specific CTL response in mice was assessed ten days after the second immunization. Spleens were collected and dissociated into single cell suspension using cell strainers (Falcon). Erythrocytes were lysed with 150mM NH_4_Cl, 1mM KHCO_3_, 0.1mM EDTA pH 7.4. Then splenocytes were washed and resuspended in RPMI 1640 containing 10% FCS (Sigma Aldrich), penicillin, streptomycin, non-essential amino acids (Gibco Life Technologies), sodium-pyruvate and 50μM β-mercaptoethanol (Gibco Life Technologies). Cell suspensions of 5_x_10^5^ and 10^6^ cells/well in a final volume of 250 μl were added to 96-well microtiter plates with nitrocellulose base (Millipore), pre-wetted with 30% ethanol and pre-coated with IFN-γ capture antibody according to manufacturer’s instructions (eBioscience). Cells were incubated for 24 hours either in presence or absence of 5 μM of synthetic HPV16 E6_41-50_, HPV16 E6_91-100_, HPV16 E6_131-140_, HPV16 E7_36-62_ peptides comprising characterized H-2b-restricted CD4^+^ (E6_41-50_, E6_91-100_) or CD8^+^ (E6_131-140_, E7_36-62_) T cell epitopes [43,44], scrambled control peptides, 5 μM influenza A H1N1 NP_311-325_, or HPV16 L1_165-173_ (all Severn Biotech Ltd.) or 2 μg/ml final concentration of *Staphylococcus aureus* enterotoxin A (SEA, Sigma Aldrich). Biotinylated anti-IFN-γ monoclonal antibodies (eBioscience) were used as second step antibodies. Finally, plates were incubated with avidin-HRP (eBioscience). Spots representing IFN-γ producing cells were developed using 3-amino-9-ethylcarbazole containing hydrogen peroxide in 0.1M sodium acetate, pH 5.0 (Dako) and counted using a dissecting microscope. Results were expressed as mean number of spot-forming cells ± SD of duplicate cultures.

To characterize the humoral immune response, sera were isolated and ELISA conducted with peptides harboring known B cell epitopes of HPV16 E6 [45,46] and E7 [47,48], i.e. HPV16 E6_11-20_, HPV16 E6_86-107_, HPV16 E6_111-120_, HPV16 E6_136-145_, HPV16 E7_36-62_, and scrambled peptide as negative control (all Severn Biotech). Whole H1N1 and H3N2 recombinant influenza A virus and HPV16 L1 VLP served as a positive controls. Peptides (1 μg/well) in PBS were coated onto Maxisorp^®^ flat-bottom 96 well plates (Nunc) for 12h at 4°C. Plates were washed and blocked for 1h with milk powder solution (0.5% in PBS) at 4°C. Plates were washed, sera diluted 1:5, 1:50 in milk powder solution and incubated for 1 h at room temperature under constant shaking. Subsequently plates were washed, incubated with HRP conjugated goat α-mouse antibody (Biorad 1:10,000) for 45 min at room temperature. Then ABTS substrate (Millipore) was added and the OD at 405 nm was determined using an ELISA-reader.

### Statistics

Statistical analysis was performed using Graph Pad Prism to evaluate p-values for the overall experiment (1-way ANOVA analysis) for ELISpot and ELISA experiments followed by Dunnet’s multiple comparison test. For TC-1 tumor cell challenge experiments p-values were calculated by Dunnett’s multiple comparison tests between control and treated groups. For statistical analysis of survival curves a Mantel-Cox test was performed.

### Ethics Statement

Animal study’s protocols were approved by the Medical University Vienna Animal Ethics Committee Board and the Austrian Federal Ministry of Science and Research (*GZ66*.*0099/0289-II/10b/2008*, *GZ66*.*0099/0057-II/3b/2013*).

## Supporting Information

S1 Fig Schematic drawing of the HPV16 E6E7 fusion proteins. The first 11 aa of NS1 were fused to wt HPV16 E6 and E7. A C-terminal FLAG-tag was added for easier detection and the construct termed 16E6E7. A mutated fusion was generated in the same manner but sequences coding for aa 73–77 and 146–151 of HPV16 E6 and aa 21–24 of HPV16 E7 were deleted as indicated to abrogate biological activity.
(TIF)

S2 Fig CTL responses to TH cell epitopes of recombinant 16E6E7 and 16E6E7m influenza viruses immunized mice. 4 mice were primed either with H1N1 16E6E7, or 16E6E7m viruses, or parental virus (delNS1), 16L1/L2-E7 VLP, or PBS (mock) and boosted 10 days later with the corresponding H3N2serotypes, or VLP or PBS as indicated. Mice were sacrificed 10 days boosting, splenocytes isolated and stimulated in triplicates for 24 h with indicated peptides, SEA or medium alone. For animals vaccinated with recombinant influenza A viruses, NP311-325 peptide served as a positive control, for mice immunized with 16L1/L2-E7 VLP, 16L1165-173 peptide was used as positive control. IFN-γ spots were counted under a light microscope and plotted as mean number ± SD. One representative experiment of two is shown.
(TIF)

S3 Fig HPV-specific humoral immunity to vaccination with recombinant 16E6E7 and 16E6E7m influenza viruses. Mice were primed s.c. with indicated recombinant or parental H1N1 viruses, or PBS (mock) and boosted 10 days later with the corresponding H3N2 serotype. Sera were isolated 10 days after the final immunization. Sera were diluted at 1:50 and incubated with peptides harbouring known antibody epitopes of HPV16 E6 and E7, whole H1N1 or H3N2 attenuated influenza A virus, or HPV16 L1 VLP attached to ELISA plates for 1 h. Secondary HRP-conjugated antibody was added for 45 min and ELISA was developed using ABTS-substrate. Colour change was monitored at 405 nm. Triplicate results are expressed as mean number ± SD. One representative experiment of two is shown.
(TIF)

S4 Fig Intratumoral administration of recombinant 16E6E7 or 16E6E7m influenza viruses elicits HPV-specific CTL responses in mice. C57BL/6 mice (n = 4 per group) were inoculated with 5x104 TC-1 cells, primed i.t. with H1N1 16E6E7, 16E6E7m viruses or parental viruses (delNS1), or PBS (mock) as soon as palpable tumours were detectable and boosted 10 days later with the corresponding H3N2 serotypes, or PBS as indicated. Mice were sacrificed 10 days after boosting, splenocytes were isolated and stimulated in triplicates for 24 h with indicated peptides, SEA or medium alone. For animals vaccinated with recombinant influenza A viruses, NP peptide served as a positive control. IFN-γ spots were counted under a light microscope and plotted as mean ± SD. One representative experiment of two is shown. Statistical p-values for 16E6E7 or 16E6E7m vaccination compared to mock are indicated as asterisks (*** p<0.001, ** p<0.01, * p<0.05).
(TIF)

S5 Fig Recombinant 16E6E7 or 16E6E7m influenza viruses infect TC-1 tumour cells **A** TC-1 cells were infected with indicated recombinant viruses for 12 h. RNA was isolated and subjected to RT-PCR to detect HPV16 E6E7 or β-actin mRNA expression, or **B** protein samples were separated by 10% SDS-PAGE and viral NP, or cellular 16E7 as loading control detected by Western blot.
(TIF)
