# Correction: Attenuated Recombinant Influenza A Virus Expressing HPV16 E6 and E7 as a Novel Therapeutic Vaccine Approach

**DOI:** 10.1371/journal.pone.0143269

**Published:** 2015-11-12

**Authors:** Christoph Jindra, Bettina Huber, Saeed Shafti-Keramat, Markus Wolschek, Boris Ferko, Thomas Muster, Sabine Brandt, Reinhard Kirnbauer

There is an error throughout the Methods section of this manuscript. The virus concentration is recorded incorrectly as 3.75 log TCID_50_. The correct concentration is 7.2 log TCID_50_.
