# Supplementary figures and images for: Attenuated Recombinant Influenza A Virus Expressing HPV16 E6 and E7 as a Novel Therapeutic Vaccine Approach
Source: PLoS One. 2015 Sep 18;10(9):e0138722. doi: 10.1371/journal.pone.0138722 (PMC4575162; doi:10.1371/journal.pone.0138722)

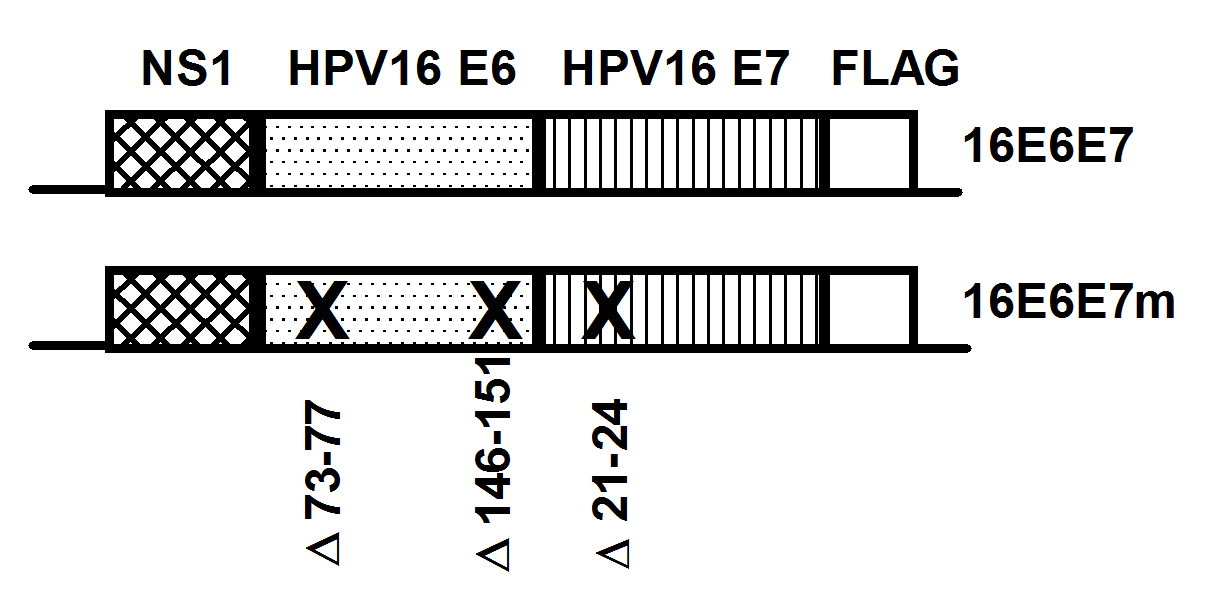

Supplement: S1 Fig — The first 11 aa of NS1 were fused to wt HPV16 E6 and E7. A C-terminal FLAG-tag was added for easier detection and the construct termed 16E6E7. A mutated fusion was generated in the same manner but sequences coding for aa 73–77 and 146–151 of HPV16 E6 and aa 21–24 of HPV16 E7 were deleted as indicated to abrogate biological activity. (TIF) [file pone.0138722.s001.tif]

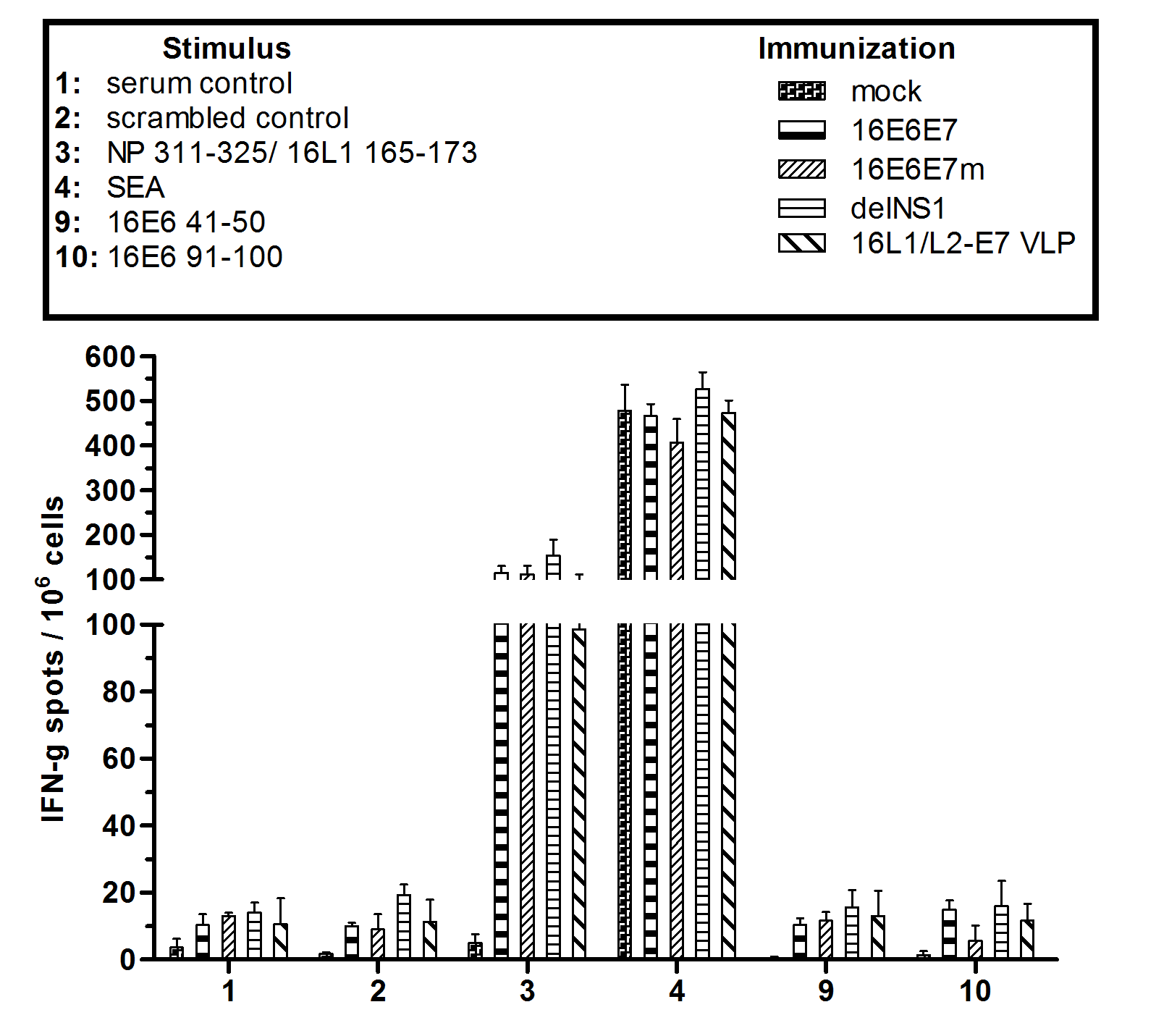

Supplement: S2 Fig — 4 mice were primed either with H1N1 16E6E7, or 16E6E7m viruses, or parental virus (delNS1), 16L1/L2-E7 VLP, or PBS (mock) and boosted 10 days later with the corresponding H3N2serotypes, or VLP or PBS as indicated. Mice were sacrificed 10 days boosting, splenocytes isolated and stimulated in triplicates for 24 h with indicated peptides, SEA or medium alone. For animals vaccinated with recombinant influenza A viruses, NP311-325 peptide served as a positive control, for mice immunized with 16L1/L2-E7 VLP, 16L1165-173 peptide was used as positive control. IFN-γ spots were counted under a light microscope and plotted as mean number ± SD. One representative experiment of two is shown. (TIF) [file pone.0138722.s002.tif]

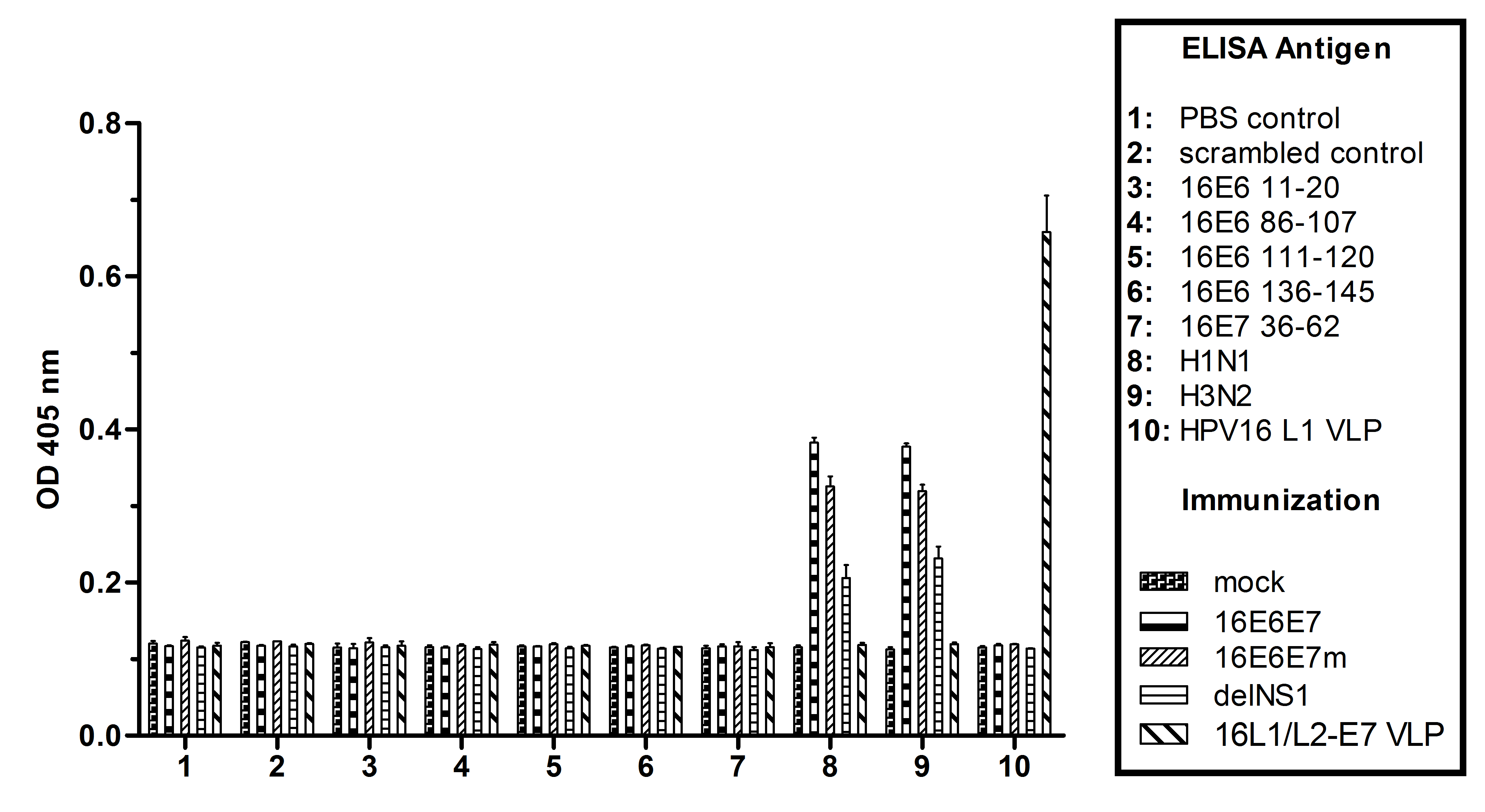

Supplement: S3 Fig — Mice were primed s.c. with indicated recombinant or parental H1N1 viruses, or PBS (mock) and boosted 10 days later with the corresponding H3N2 serotype. Sera were isolated 10 days after the final immunization. Sera were diluted at 1:50 and incubated with peptides harbouring known antibody epitopes of HPV16 E6 and E7, whole H1N1 or H3N2 attenuated influenza A virus, or HPV16 L1 VLP attached to ELISA plates for 1 h. Secondary HRP-conjugated antibody was added for 45 min and ELISA was developed using ABTS-substrate. Colour change was monitored at 405 nm. Triplicate results are expressed as mean number ± SD. One representative experiment of two is shown. (TIF) [file pone.0138722.s003.tif]

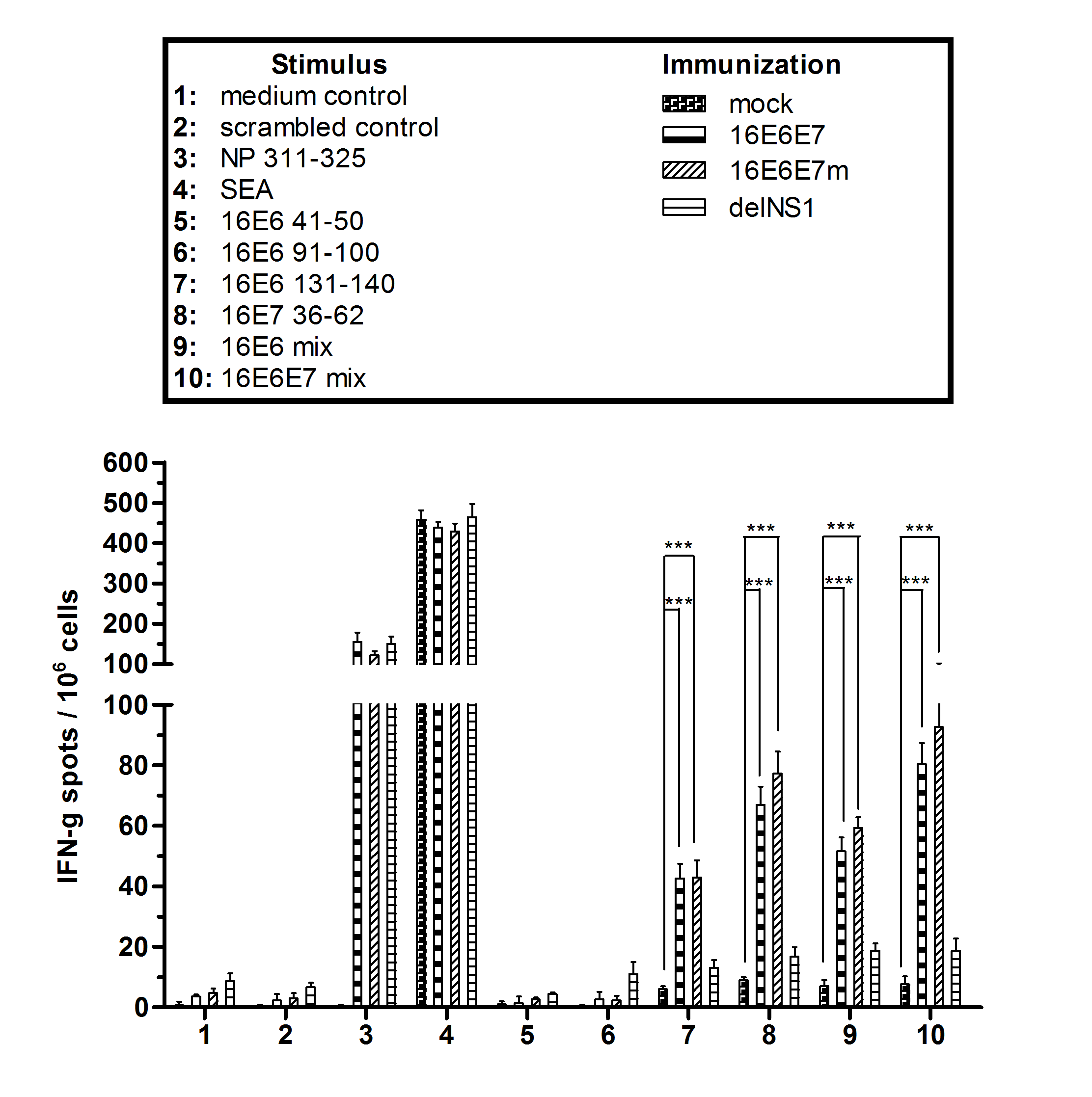

Supplement: S4 Fig — C57BL/6 mice (n = 4 per group) were inoculated with 5x10^4^ TC-1 cells, primed i.t. with H1N1 16E6E7, 16E6E7m viruses or parental viruses (delNS1), or PBS (mock) as soon as palpable tumours were detectable and boosted 10 days later with the corresponding H3N2 serotypes, or PBS as indicated. Mice were sacrificed 10 days after boosting, splenocytes were isolated and stimulated in triplicates for 24 h with indicated peptides, SEA or medium alone. For animals vaccinated with recombinant influenza A viruses, NP peptide served as a positive control. IFN-γ spots were counted under a light microscope and plotted as mean ± SD. One representative experiment of two is shown. Statistical p-values for 16E6E7 or 16E6E7m vaccination compared to mock are indicated as asterisks (*** p<0.001, ** p<0.01, * p<0.05). (TIF) [file pone.0138722.s004.tif]

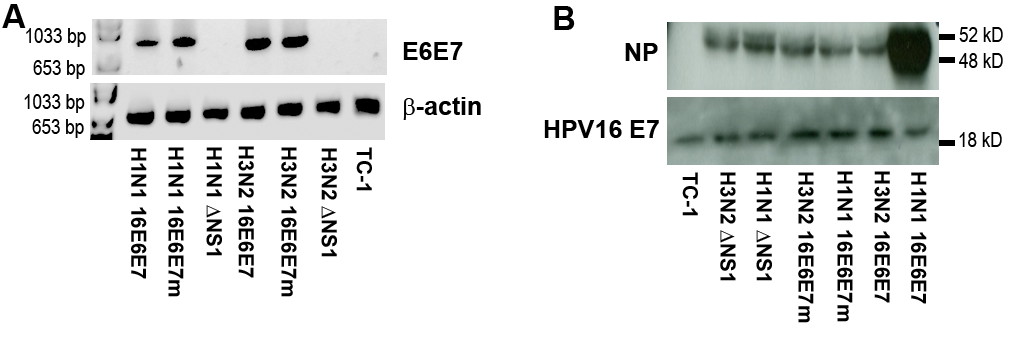

Supplement: S5 Fig — A TC-1 cells were infected with indicated recombinant viruses for 12 h. RNA was isolated and subjected to RT-PCR to detect HPV16 E6E7 or β-actin mRNA expression, or B protein samples were separated by 10% SDS-PAGE and viral NP, or cellular 16E7 as loading control detected by Western blot. (TIF) [file pone.0138722.s005.tif]
